# Both high and low pre-infection glucose levels associated with increased risk for severe COVID-19: New insights from a population-based study

**DOI:** 10.1371/journal.pone.0254847

**Published:** 2021-07-22

**Authors:** Michal Shauly-Aharonov, Asher Shafrir, Ora Paltiel, Ronit Calderon-Margalit, Rifaat Safadi, Roee Bicher, Orit Barenholz-Goultschin, Joshua Stokar

**Affiliations:** 1 Braun School of Public Health and Community Medicine, Hebrew University of Jerusalem, Jerusalem, Israel; 2 The Jerusalem College of Technology, Jerusalem, Israel; 3 Division of Medicine, Meuhedet Health Services, Tel Aviv, Israel; 4 Faculty of Medicine, Hebrew University of Jerusalem, Jerusalem, Israel; 5 Department of Gastroenterology, Hadassah Medical Center, Jerusalem, Israel; 6 Department of Hematology, Faculty of Medicine, Hadassah-Hebrew University Medical Center, Ein Karem Campus, Hebrew University of Jerusalem, Jerusalem, Israel; 7 Division of Medicine, Liver and Gastroenterology Unit, Hadassah University Medical Center, Jerusalem, Israel; 8 Finance Division, Department of Information, Meuhedet Health Services Tel Aviv, Israel; 9 Department of Obstetrics & Gynecology and Endocrinology Institute, Shaare Zedek Medical Center, Jerusalem, Israel; 10 Department of Endocrinology, Hadassah Medical Center, Jerusalem, Israel; The Ohio State University College of Medicine, UNITED STATES

## Abstract

**Importance:**

Patients with diabetes are known to be at increased risk for infections including severe coronavirus disease 2019 (COVID-19) but the relationship between COVID-19 severity and specific pre-infection glucose levels is not known.

**Objective:**

To assess the differential effects of pre-infection glucose levels on the risk for severe COVID-19 amongst patients with and without diabetes.

**Design:**

Population based historical cohort study.

**Setting:**

National state-mandated HMO.

**Patients:**

All adult patients with a positive SARS-COV2 test between March-October 2020.

**Exposure:**

Recent fasting blood glucose (FBG) and glycated hemoglobin (HBA1C), age, gender, body mass index (BMI) and diagnoses of diabetes, hypertension, ischemic heart disease.

**Outcome:**

Risk for severe COVID-19, defined as resulting in ≥10 hospitalization days, ICU admission or death.

**Results:**

37,121 patients with a positive SARS-COV2 test were identified; 707 defined as severe (1.9%). Unadjusted risk factors for severe disease were age (OR = 1.1 for every year increase; 95% CI 1.09–1.11, p < 0.001), male gender (OR = 1.34, 95% CI 1.06–1.68, p = 0.012); BMI (OR = 1.02 for 1 kg/m^2^ increase, 95% CI 1.00–1.04, p = 0.025). Controlling for these factors, we found an association between pre-infection FBG and the risk of severe COVID-19, with a differential effect in patients with and without a diagnosis of diabetes. For patients without diabetes, elevated FBG in the pre-diabetes range (106–125 mg/dl) was associated with severe COVID-19 (OR 1.55 95% CI 1.04–2.26 p = 0.027). For patients with a diagnosis of diabetes, we found a J-shaped association between pre-infection glucose control and the risk for severe COVID-19 where the lowest risk for was for patients with FBG 106–125 mg/dl; the risk increased with higher pre-infection glucose levels but strikingly also for patients with a low pre-infection FBG (<100mg/dl) or HbA1C (<5.7%).

**Conclusions and relevance:**

Elevated pre-infection blood glucose is a risk factor for severe COVID-19 even in non-diabetics. For patients with a diagnosis of diabetes both high as well as low pre-infection glucose levels are risk factors for severe COVID-19. Further research is required to assess whether these associations are causal, but we believe these findings can already have clinical implications for COVID-19 risk assessment and stratification.

## Introduction

Patients with diabetes are known to be more susceptible to infections. Moreover, once infected they have a higher risk of complications [1]. As with previous corona virus outbreaks [2, 3], diabetes mellitus was consistently shown to be a predisposing factor for severe COVID-19. Several large cohort studies demonstrated a clear association between preexisting diabetes, HbA1C as well as hyperglycemia upon admission, and COVID-19 related mortality [2, 3]. A study from Israel found pre-infection elevated HbA1C to be associated with increased risk of hospitalization [4]. Not surprisingly, diabetes was also found to be an important risk factor for ICU admissions [5]. Other studies described the association between blood glucose control among patients with well-established diabetes and COVID-19 outcome, focusing on hyperglycemia upon admission and HbA1C>7.5% among hospitalized patients [6, 7], as well as for newly diagnosed diabetes [8]. For non-diabetics, a j-shaped association between blood glucose at hospital admission and COVID-19 outcome was found, with the best outcome in the group with FBG of 85–94 mg/dl [9]. It was suggested that clinicians strive for glucose levels below 180 mg/dl in diabetic patients admitted to hospital due to COVID-19 infection [10], similarly to the target glucose levels in other hospitalized patients [11].

Though related, it is important to distinguish between the abnormal glucose levels prior to COVID-19 infection and those found during the acute infection phase. Even patients without a prior diagnosis of diabetes may suffer from “stress hyperglycemia” during an acute illness [12]. Such patients are at higher risk for acute complications of their disease as well as for future development of diabetes [13].

Several pathophysiological mechanisms have been proposed to explain the increased severity of COVID-19 amongst patients with diabetes or hyperglycemia [14]. Obviously, any anaylsis of such associations must control for other confounding risk factors prevalent amongst patients with diabetes such as obesity, increased age, and other co-morbid conditions [15].

The purpose of this study was to search for risk factors of severe COVID-19 infection, that might be identified before infection, and that could be amenable to modification or improvement based on physicians’ awareness and public education as well as to be considered during risk stratification and vaccine prioritization. To our knowledge, no population-based study has been published to date regarding the association between pre-infection glucose levels and risk of severe COVID-19, in patients with and without diabetes.

## Methods

We conducted a historical cohort study in the setting of Meuhedet health maintenance organization (HMO). Meuhedet HMO is the third largest state mandated healthcare provider in Israel, serving over 1,200,000 individuals throughout the country. Meuhedet’s comprehensive and integrated computerized database includes real time input from all physician visits, medical diagnoses, laboratory results, hospitalizations and dispensing data on prescription medications and nonprescription, over-the-counter medications. Data from EMRs for all insured individuals aged 18 and above who underwent a SARS-COV-2 PCR test from March 1, 2020 to October 31, 2020 were extracted. The laboratory confirmation of SARS-COV-2 infection was defined as a positive result of a real-time PCR assay from nasal and pharyngeal swabs, in accordance with World Health Organization guidelines [16]. In cases where a patient had multiple tests during this time-period, the first positive test was considered the index test. When all tests were negative, the first SARS-COV-2 test was considered the index test. SARS-COV-2 test results from outside the HMO (e.g., hospitals) were also included.

All HMOs in Israel define three sectors of the Israeli society based on the location of the clinic. These are: Arab, ultra-orthodox Jewish, and general Jewish. COVID-19 prevalence differs among these groups [17], therefore, this variable was included as a potential risk factor. Additional data extracted from EMRs included patient age, gender, and body mass index (BMI). To avoid confounding from the effect of the acute infection, results of the most recent FBG test from the prior year performed at least 30 days before the SARS-COV-2 index test were included in our data set, as well as glycosylated hemoglobin (HbA1C). In addition, major medical diagnoses documented at any point prior to SARS-COV-2 testing (e.g., hypertension, diabetes, hyperlipidemia, hypothyroidism) were recorded. A diagnosis of diabetes was defined based on ICD-9 code 250 and its sub-divisions.

Outcomes considered in this analysis included: 1. number of days a patient was hospitalized during the two months following a positive SARS-COV-2 test 2. any hospitalization in the ICU, and 3. death. We considered severe infection as any event of death, ICU admission or hospitalization of ten days or more following infection with SARS-COV-2. The hospital system in Israel is based on mandatory state issued health insurance, thus length of hospitalization and admission to ICU can be viewed as objective measures of disease severity as they are not significantly affected by financial incentives or issues of health insurance coverage.

The study was approved by the research ethics committee (IRB) of Meuhedet HMO.

### Statistical analysis

Statistical analysis was performed using R software [18]. The packages ‘ggplot2’, ‘dplyr’, ‘Matching’ and ‘tidy’ were used in addition to the default R packages. Categorical variables were summarized as counts and percentages. Continuous variables were summarized as means and standard deviations (SD). Univariate analysis was performed using Chi-squared test to compare categorical variables, and t-test to compare means of continuous variables. Logistic regression was performed to assess the effect of prior FBG on severe COVID-19, controlling for patient characteristics that had been found in the literature to be potential confounders. Patients with missing data in one (or more) of the confounders were excluded from the regression. Variables with a p-value of less than 0.2 on univariate analysis were considered as potential confounders (and thus were included in the covariate selection process in the multivariate analysis). These included age, gender, BMI, prior diagnosis of ischemic heart disease (IHD), hypertension and diabetes, and the sector to which the clinic belongs (Arab / ultra-orthodox Jewish / general Jewish). Given the clinical likelihood that the presence of diabetes modifies the association between prior FBG and severe infection, we tested the significance of the interaction between these factors in the logistic regression mentioned above. P-value of less than 0.05 was considered statistically significant in all analyses. In addition to p-values, effect sizes are also presented; for continuous variables, these represent the difference in the averages divided by the standard deviation when one combines all the measurements (providing an estimate of the number of standard deviations the means are apart), and for categorical (yes/no) variables, it is the difference in the proportions divided by $\sqrt{p\left(1-p\right)}$, where *p* is the proportion in the combined sample. In the case of the logistic regressions, odds ratios (OR) and their corresponding 95% confidence intervals (CI) are given. A 1:1 matched case-control analysis was performed to assess the differences in prior FBG between cases of severe COVID-19 and controls (individuals with non-severe COVID-19), while matching on age, gender, overweight (BMI>25) and ischemic heart disease. A greedy-match algorithm was used. McNemar test and Wilcoxon’s singed-rank test were performed on these paired data, as well as a logistic regression.

## Results

Between 1.3.2020 and 30.10.2020, 322,808 patients enrolled in Meuhedet HMO had at least one RT-PCR test for SARS-COV-2. Of these, 214,470 (66.4%) were adults, 18 years of age and older. Of the adults, 37,121 (16.7%) had a positive test. Comparisons between patients who tested positive and negative are presented in S1 Appendix. Among adult patients who tested positive for SARS-COV-2, 707 (1.9%) had a severe infection. Of these, 244 died, 188 were hospitalized in ICU, and 538 were hospitalized for 10 days or more (non-mutually exclusive). Patients who had severe infection tended to be older (68.18 [17.9] vs. 36.66 years [15.6]), obese (BMI>30 kg/m^2^, 42.8% vs. 24.6%), have a diagnosis of diabetes (36.5% vs. 6.2%), IHD (18.1% vs. 2.1%) and hypertension (28.9% vs. 4.0%). Other characteristics are shown in Table 1.

**Table 1:** Characteristics of patients with severe COVID-19 infection, compared to patients with a non-severe infection.

	Severity of COVID-19 infection		Effect Size
	non-severe	severe	
**N**	36414	707	
**Age (mean (SD))**	36.66 (15.64)	68.18 (17.89)	1.876
**Male Gender (%)**	20756 (57)	403 (57)	0.015
**BMI > 30 (%)**	6948 (24.6)	236 (42.8)	0.394
**Sector (%)**			0.447
Arabs	5866 (16.1)	137 (19.4)	
General Jews	10756 (29.5)	335 (47.4)	
Ultra-orthodox Jews	19792 (54.4)	235 (33.2)	
**Prior Diabetes Type2 (%)**	2244 (6.2)	258 (36.5)	0.797
**Prior Ischemic Heart Disease (%)**	754 (2.1)	128 (18.1)	0.552
**Smoking (%)**	2897 (8.0)	42 (5.9)	0.079
**Hypertension (%)**	1443 (4.0)	204 (28.9)	0.714

**Fig 1:**
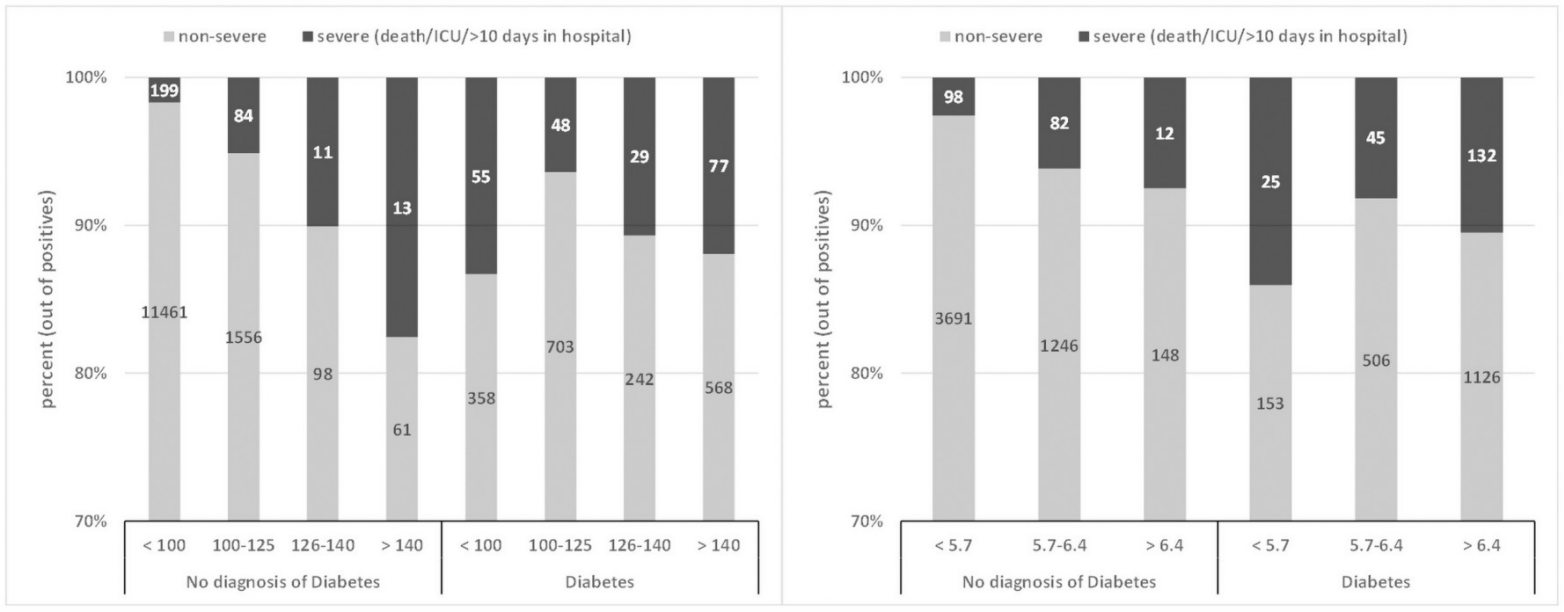
**Left (A)**: Percentage of severe COVID-19 (death/ICU/≥10 hospitalization days) given prior fasting glucose range (mg/dl), for patients with no diagnosis of diabetes vs. patients with a diagnosis of diabetes. The numbers on the bars represent the number of patients in each sub-category, whereas the inner partition into red and blue represents the conditional distribution of severity, given the patients are in the specific range of FBG. **Right (B):** Percentage of severe COVID-19 (death/ICU/≥10 hospitalization days) given prior HbA1C, for patients with no diagnosis of diabetes (left) vs. patients with a diagnosis of diabetes (right).

**Fig 2:**
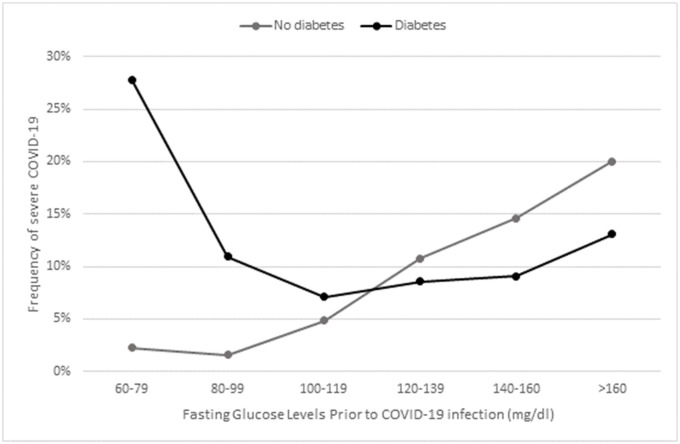
Frequency of severe COVID-19 (death/ICU/ hospitalization ≥10 days) by pre-infection fasting blood glucose, among patients with (in black) and without (in grey) a diagnosis of diabetes.

Of the adult patients with a positive test for SARS-COV-2, 15,563 (42.0%) had a recent fasting glucose test and 7,264 (19.6%) had an HbA1C test recorded in the HMO prior to the PCR test. Overall, fasting glucose and HbA1C were both significantly higher in patients with severe infection (FBG: 114.57 mg/dl vs. 94.87 mg/dl, p<0.001, HbA1C: 6.56% vs. 5.85%, p<0.001). In a univariate logistic regression analysis on the 15,563 positive patients with FBG value, we found that age, gender, BMI, sector, prior diagnosis of IHD, hypertension and diabetes were potential confounders (i.e., p<0.2), thus included as covariates in the multivariate analysis. Of the 15,563 infected adults with an FBG value, 12,481 had a recorded value for BMI. In a multivariate logistic regression on these 12,481 patients, there were significant associations between the risk of severe COVID-19 and: older age (OR = 1.1 for 1 year increase, 95% CI 1.09–1.11, p < 0.001); male gender (OR = 1.34, 95% CI 1.06–1.68, p = 0.012); and BMI (OR = 1.02 for 1 kg/m^2^ increase, 95% CI 1.00–1.04, p = 0.025). Ischemic heart disease showed an effect of borderline significance (OR = 1.33, CI 0.99–1.78, p = 0.058). The interaction effect between diabetes and FBG was highly significant (p = 0.0004), and we therefore performed separate analyses for patients with a diagnosis of diabetes and for those without. These analyses are described in the following sub-sections. Figs 1A and 2 demonstrate this interaction clearly, as they show a different relationship between FBG and severe COVID-19 for patients diagnosed with diabetes and for patients with no such diagnosis. A similar interaction was seen for HbA1C (see Fig 1B).

### Association between glucose levels and severe covid-19 in patients without a diagnosis of diabetes

In a sub-analysis of patients without a documented diagnosis of diabetes (n = 10,763), FBG was significantly higher in patients who had a severe infection (96.36 vs. 89.02, p<0.001). A similar association was seen for HbA1C (5.66 vs. 5.40, p<0.001). In a multivariate logistic regression controlling for age, gender, sector, BMI, presence of IHD and HTN, FBG above 105 mg/dl was associated with an increased risk of severe disease, compared to FBG≤105 mg/dl. This risk further increased as fasting glucose levels rose; specifically, a significantly higher risk was observed for: FBG of 106–125 mg/dl (OR 1.55 95% CI 1.04–2.26 p = 0.027), FBG of 126–140 mg/dl (OR 2.1, 95% CI 0.86–4.55 p = 0.07), and FBG>140 mg/dl (OR 2.6, 95% CI 1.05–5.91 p = 0.029). The entire output of the regression model appears in Table 2.

**Table 2:** Regression results for severe COVID-19 among non-diabetics (n = 10,763).

COVARIATE	Odds-ratio	Std.error	p.value	95% Confidence interval	
**Age**	1.103	0.005	0.000	1.092	1.114
**gender Male**	1.488	0.147	0.007	1.117	1.985
**GLUCOSE GROUP (reference group: glucose ≤ 105 mg/dl)**					
**glucose 106–126**	1.548	0.197	0.027	1.040	2.257
**glucose 127–140**	2.104	0.420	0.077	0.863	4.554
**glucose > 140**	2.601	0.438	0.029	1.048	5.910
**SECTOR (reference group: Arabs)**					
**General Jews**	0.860	0.197	0.443	0.588	1.274
**Ultra-orthodox jews**	0.630	0.214	0.031	0.416	0.962
**BMI**	1.020	0.013	0.121	0.994	1.047
**Prior Ischemic Heart Disease**	1.232	0.220	0.343	0.791	1.876
**Prior Hypertension**	1.171	0.179	0.380	0.818	1.654

### Association between glucose and severe COVID-19 infection in diabetics

Figs 1A and 2 demonstrate that for patients with a diagnosis of diabetes (n = 1718), the lowest percentage of severe infections was observed in the group with pre-infection FBG of 106–125 mg/dl. The risk of severe infection increased as glucose levels rise above 126 mg/dl, while those with FBG≤105 mg/dl have even more increased risk. This finding was statistically significant in a multivariate logistic regression controlling for age, gender, sector, BMI, HTN, and IHD; Comparing to the (reference) group of FBG 106–126 mg/dl, the group with FBG≤105 mg/dl had a higher risk (OR 2.034, 95% CI 1.215–3.474, p = 0.008); the group with FBG 127–140 mg/dl also had a higher risk (OR 2.567, 95% CI 1.339–4.882, p = 0.004), and the group with FBG>140 had the highest risk (OR 3.321, 95% CI 2.033–5.577, p<0.0001). Table 3 shows the entire output of the regression model.

**Table 3:** Regression results for severe COVID-19 among diabetics (n = 1718).

COVARIATE	Odds-Ratio	Std.error	p.value	95% Confidence interval	
**Age**	1.097	0.009	0.000	1.079	1.117
**Gender Male**	1.104	0.191	0.604	0.761	1.612
**FASTING GLUCOSE (reference group: 106–126 mg/dl)**					
**glucose ≤ 105**	2.034	0.267	0.008	1.215	3.474
**glucose 127–140**	2.567	0.328	0.004	1.339	4.882
**glucose > 140**	3.321	0.257	0.000	2.033	5.577
**BMI**	1.026	0.017	0.126	0.992	1.061
**SECTOR (reference group: Arabs)**					
**Jeneral Jews**	1.472	0.232	0.095	0.939	2.338
**Ultra-orthodox jews**	1.260	0.248	0.351	0.777	2.060
**Prior Ischemic Heart Disease**	1.450	0.207	0.072	0.961	2.162
**Prior Hypertension**	1.205	0.190	0.324	0.828	1.743

To reinforce this finding, a matched case-control analysis was performed among patients with a diagnosis of diabetes with fasting glucose lower than 140 mg/dl. Patients with severe infection (cases, n = 97) were matched 1:1 to patients with non-severe infection (control, n = 97) by age, gender, obesity (BMI>30 kg/m^2^) and presence of IHD. The proportion of patients with FBG<100 mg/dl was significantly higher in the group with severe infection than in the group with a non-severe infection (45.4% vs. 29.9%, McNemar p = 0.038). A univariate logistic regression showed similar results (p = 0.027, OR 1.95 95% CI 1.08–3.54). The marginal means of the variables that were matched are given in Table 4.

**Table 4:** Characteristics of diabetic patients in the matched case-control analysis, by severity of COVID-19.

	Non-severe COVID-19	Severe COVID-19	P-value
**N**	97	97	
**Age (mean (SD))**	72.44 (12.94)	73.45 (13.66)	0.598
**Male Gender (%)**	56 (57.7)	56 (57.7)	1.000
**Obesity (%)**	83 (85.6)	83 (85.6)	1.000
**Prior IHD (%)**	31 (32.0)	31 (32.0)	1.000
**FBG<100mg/dl (%)**	29 (29.9)	44 (45.4)	0.038

FBG<100 mg/dl was the investigated proportion; all other characteristics were matched between the severe and non-severe.

## Discussion

Many of the previous reports of risk factors for severe COVID-19 were derived from hospital EMRs. Such reports obviously contain important information but need to be interpreted cautiously as the hospital setting introduces a selection bias by-definition. Other reports used crude rates without adjustment for additional risk factors and co-morbidities [15]. Our data are derived from a national state-mandated HMO database reflecting the entire range of Israeli population tested for SARS-COV-2 over a relatively long period including information on demographics and additional risk factors. Regarding socio-demographics, we found a lower risk for severe disease in the ultra-orthodox sector, though this was driven only by length of hospitalization. Thus, we believe this finding stems from unique home-based care programs initiated in this sector allowing for shorter hospitalizations.

For the other risk factors, our results are in-line with previous reports of risk factors for severe COVID-19, with the strongest associations found for age, obesity, and diabetes. However, we extend the findings to pre-infection glycemic levels, and not just those on admission to hospital. Interestingly, we did not find an association between severe COVID-19 and smoking or HTN when controlling for known risk factors. The adjusted OR for severe infection amongst patients with diabetes versus those without diabetes seems in-line with the OR found amongst patients with diabetes for other severe infections [1]. Obviously, it is hard to separate the effect of hyperglycemia on the risk for severe infections in general from that specific to COVID-19.

The central finding of this study was a significant effect of prior FBG levels on COVID-19 severity, both among patients with a diagnosis of diabetes and those without. For patients without a diagnosis of diabetes, prior FBG in the pre-diabetes range (FBG 100–125, HbA1c 5.7–6.4) was associated with an increased risk for severe disease relative to those in the normoglycemic range (FBG <100). However, the increase was statistically significant only for FBG>105 mg/dl. Patients without a diagnosis of diabetes with prior FBG >125 or HbA1C>6.4 showed even higher risk for severe disease, and patients with FBG above 140 mg/dl were observed to be at the highest risk, although not diagnosed with diabetes. The American diabetes association and the local Israeli guidelines require two abnormal glucose tests for the diagnosis of diabetes, so these patients most probably have yet to be or perhaps undiagnosed diabetes. The fact that they show similar increased risk for severe COVID-19 as patients with overt diabetes may not be surprising but does have practical implications. Both at the individual patient care level as well as at the public health level, such patients should be sought out and considered high-risk for severe COVID-19 together with patients with overt diabetes. To strengthen the results of this study, we performed the same analysis but considered patients with FBG >125 mg/dl together with patients with a diagnosis of diabetes. The results were similar, both for diabetic and non-diabetic groups, both in terms of statistical significance and effect size.

Amongst patients with a diagnosis of diabetes, we found an increased risk for severe COVID-19 for FBG>125 mg/dl, which further increased with FBG above 140 mg/dl. However, the remarkable finding was the increased risk for severe COVID-19 amongst patients with diabetes with a prior low FBG (<100mg/dl) or HbA1C (<5.7%). This association remained significant after adjustment for age, BMI, gender, IHD, hypertension and sector, as well as in a separate matched case-control analysis. This finding is compatible with results of several other large studies that found a J shaped association between prior glycemic control and risk for severe infections [19, 20], as well as for all-cause mortality [21]. One possible explanation for this finding is that this group contains more patients treated with insulin which can lead to a lower blood glucose and HbA1C. As insulin therapy is usually reserved for advanced type 2 or type 1 diabetes, this may reflect patients with increased diabetes severity. Unfortunately, data on patient medications or specific type of diabetes were not available to us to test this hypothesis. However, other studies have not found a significant difference between type 1 and type 2 diabetes on the risk of severe COVID-19 [22]. An alternative additional explanation could be that these patients are being aggressively “over-treated” for their diabetes. It is well documented that recurrent hypoglycemia associated with “tight control” of blood glucose is a significant risk factor for morbidity and mortality among patients with diabetes [23, 24]. Further dedicated research is required to understand this novel finding, though we believe it too can already have practical implications. Despite their seemingly “normal” glucose profile, the increased risk seen for severe COVID-19 in this group should not be overlooked.

Due to its observational design, causality could not be directly assessed in our study and despite multiple-adjustments and matching, hidden potential confounders can never be fully controlled for. Additionally, our research design and data availability did not allow for testing of the various hypotheses regarding the causal relationship between hyperglycemia and COVID-19 severity. We chose to include all-cause mortality instead of COVID-19 specifc mortality in defining severe COVID-19 as we believe this to be a relevant clinical outcome for assessment of COVID-19 risk for groups as well as for individual patients. Additional limitations of this study include the lack of differentiation between types of diabetes as well as the duration of disease. The use of a single FBG measurement does not completely capture an individual patient’s blood glucose pattern, but as we found a similar pattern using HBA1C the study’s main observation seems to be valid. The major strength of the study stems from its large numbers and population-based design, strengthening the generalizability of the findings.

In summary, we found that both a diagnosis of diabetes as well as elevated FBG are significant risk factors for severe COVID-19. Specific notice should be taken to patients without an official diagnosis of diabetes with elevated FBG or HbA1C as well as to patients with diabetes and low FBG or HbA1C, who are also at increased risk for severe COVID-19.

## Supporting information

S1 Appendix (DOCX)
