# Supplementary material for: Both high and low pre-infection glucose levels associated with increased risk for severe COVID-19: New insights from a population-based study
Source: PLoS One. 2021 Jul 22;16(7):e0254847. doi: 10.1371/journal.pone.0254847 (PMC8297851; doi:10.1371/journal.pone.0254847)
Supplement: S1 Appendix — (DOCX) [file pone.0254847.s001.docx]

**Appendix 1**. Characteristics of adult patients with PCR testing for SARS-COV-2 from 1.3.2020 to 30.10.2020.

|  | **COVID-19 TEST RESULT** | |  |
| --- | --- | --- | --- |
|  | **negative** | **positive** | *Effect Size* |
| **N** | 177349 | 37121 |  |
| **Age (mean (SD))** | 41.28 (17.81) | 37.26 (16.26) | 0.236 |
| **Sex = male (%)** | 82919 (46.8) | 21002 (56.6) | 0.198 |
| **BMI (mean (SD))** | 26.43 (5.49) | 26.45 (5.85) | 0.002 |
| **Sector (%)** |  |  | 0.541 |
| Arabs | 31970 (18.0) | 6003 (16.2) |  |
| General Jews | 93334 (52.6) | 11091 (29.9) |  |
| Ultra-orthodox Jews | 52045 (29.3) | 20027 (54.0) |  |
| **Prior Diabetes Type2 (%)** | 13631 (7.7) | 2502 (6.7) | 0.037 |
| **Prior Ischemic Heart Disease (%)** | 6567 (3.7) | 882 (2.4) | 0.077 |
| **Smoking (%)** | 27160 (15.3) | 2939 (7.9) | 0.232 |
| **Prior Hypertension (%)** | 11762 (6.6) | 1647 (4.4) | 0.096 |
